# Workflow optimization in a clinical laboratory using Lean management principles in the pre-analytical phase

**DOI:** 10.5937/jomb0-26055

**Published:** 2021-01-26

**Authors:** Pablo Letelier, Neftalí Guzmán, Gustavo Medina, Luis Calcumil, Pamela Huencho, Jonathan Mora, Francisco Quiñones, Jorge Jara, Cristóbal Reyno, Jorge G. Farías, Belén Lisandra Herrera, Priscilla Brebi, Ismael Riquelme, Martín Andrés San

**Affiliations:** 1 Universidad Católica de Temuco, Facultad de Ciencias de la Salud, Departamento de Procesos Diagnósticos y Evaluación, Precision Health Research Laboratory, Temuco, Chile; 2 Hospital Dr. Hernán Henríquez Aravena, Clinical Laboratory, Temuco, Chile; 3 Universidad de La Frontera, Scientific and Technological Bio-resource Nucleus, Genomic Unit; 4 Universidad de La Frontera, Faculty of Engineering and Science, Department of Chemical Engineering, Temuco, Chile; 5 Universidad de La Frontera, Scientific and Technological Bioresource Nucleus (BIOREN), Center for Excellence in Translational Medicine (CEMT), Laboratory of Integrative Biology (LIBi), Temuco, Chile; 6 Universidad Autónoma de Chile, Facultad de Ciencias de la Salud, Instituto de Ciencias Biomédicas, Chile

**Keywords:** clinical laboratory, Lean methodology, preanalytical phase, pre-analitička faza, Lean metodologija, klinička laboratorija

## Abstract

**Background:**

The application of the Lean methodology in clinical laboratories can improve workflow and user satisfaction through the efficient delivery of analytical results. The purpose of this study was to optimise delivery times of the test results at a clinical laboratory, using Lean management principles in the pre-analytical phase.

**Methods:**

A prospective study with a quasi-experimental design was implemented. Staff functions were restructured and sample flows were modified. Delivery times of clinical results (glucose and haematocrit; 6648 data) from the Medicine and Adult Emergency services for years 2017 and 2018 were compared.

**Results:**

A reduction (*p < 0.05*) in turnaround times in the delivery of glucose test results at the adult emergency service was observed (84 to 73 min, 13%, pre and post). In addition, there was a non-significant reduction in the turnaround times for glucose (Medicine) and haematocrit in both services. In the analytical and post-analytical phase (not intervened), an increase in turnaround times was observed in some cases.

**Conclusions:**

Other studies have indicated that the application of the Lean methodology in clinical laboratories improves workflow, increasing effectiveness and efficiency. This study showed an improvement in the delivery time of test results (glucose - Emergency), giving rise to a culture of cooperation and continuous improvement. It would, however, be essential to address the management model integrating the analytical and post-analytical phases.

## Abstract (sr)

**Uvod:**

Primena Lean metodologije u kliničkim laboratorijama može da poboljša protok i funkcionisanje efikasnosti laboratorije u svrhu dobijanja analitičkih rezultata. Svrha ovog proučavanja je da se optimizuje vreme isporuke rezultata ispitivanja u kliničkoj laboratoriji primenom principa Lean-menadžmenta u pre-analitičkoj fazi.

**Metode:**

Primenjena je prospektivna studija kvazi-eksperimentalnog modela. Funkcionisanje osoblja je prilagođeno i pojednostavljen je protok uzoraka. Vreme isporuke kliničkih rezultata (glukoze i hematokrita) iz Kliničkog i Urgentnog odeljenja za odrasle u periodu od 2017 do 2018.

**Rezultati:**

Nađeno je smanjenje (p < 0,05) obrtnog vremena pri isporuci rezultata za glukozu u odeljenje urgentne službe (84 do 73 min, 13%, pre i post). Dodatno nije bilo značajnog smanjenja obrtnog vremena za izdavanje rezultata za glukozu i hematokrit za kliničko odeljenje. U analitičkoj i post-analitičkoj fazi uočeno je izvesno povećanje obrtnih vremena u izvesnim slučajevima.

**Zaključak:**

Druga proučavanja koja su primenjivala Lean metodologiju u kliničkim laboratorijama ukazuju na poboljšanje protoka, efikasnosti i efektivnosti. Ovo izučavanje ukazuje na poboljšanje vremena isporuke rezultata ispitivanja (glukoza - hitnoj službi), ukazujući na stalnu mogućnost poboljšanja. Potrebno je ukazati da je neophodna primena menadžmenta na integrisanu analitičku i post-analitičku fazu.

## Introduction

A clinical laboratory is a fundamental unit in the support of diagnostic, prognostic, treatment control and prevention of different human pathologies. Its main role is to produce reliable, reproducible and timely results to assist in the making of clinical decisions [1]. Its operation requires maintaining an efficient and coordinated workflow with other services, under a strict system that will ensure the quality of its results in the pre-analytical, analytical and post-analytical phases [2].

At present efficiency has improved and error ratios have decreased, both in the analytical and postanalytical phases, as a result of the standardisation of *in vitro* methodologies, advances in instrumentation, availability of qualified staff and the implementation of laboratory information systems [3], ensuring traceability of the clinical samples and providing valuable management information in support of continuous improvement [4]. However, the pre-analytical phase is associated with a greater number of errors [5] [6], constituting one of the most complex phases to control because it has a series of variables and several critical points that require improvement (biological, environmental, and technical factors) [7]. This phase can be divided into an extra-laboratory stage (analysis ordering, patient preparation, specimen collection, transport and temporary storage) [6] and an intra-laboratory stage (reception, admittance and labelling of samples, centrifugation, distribution and preparingaliquoting specimens for analysis) [7] [8].

Lean is a management model derived from the Toyota Production System, first implemented at the Toyota Japanese car factory [9] [10], which emphasizes the elimination of waste and non-value-added steps in a setting of limited resources, maintaining patient satisfaction [11]. The pillars of this model are continuous improvement and respect of individuals, and its work philosophy aims at using human staff and available resources to improve user services. The available evidence supports the idea that the application of the Lean methodology in health institutions and particularly in clinical laboratories can improve workflow and performance [12] [13] [14] [15]. With regard to improvement models incorporated into the pre-analytical phase, there is currently scant evidence to support that the models have had much impact on the reduction of turnaround times in the delivery of clinical test results.

The goals of the project were to improve turnaround times (TT) of laboratory results, maximise workflow and eliminate waste, incorporating some principles of the Lean management model in the preanalytical phase of a clinical laboratory. Before improving the process, we also made sure that other services and units were informed and available to help with changes in workflow.

## Materials and Methods

### Study design

This study was a prospective, before-after analysis of process improvements in the clinical laboratory of the Dr. Hernán Henríquez Aravena Hospital in Temuco, Chile. This high-complexity laboratory performs analyses in the areas of haematology and haemostasis, clinical biochemistry, microbiology, tuberculosis, serology, flow cytometry and molecular biology. It carries out approximately 3.5 million tests per year and is furnished with a Labcore Laboratory Information System (LIS), which allows it to verify the results of the analysers and transmit them to the computerised record system, maintaining the traceability of the clinical samples. The intra-laboratory pre-analytical section of the hospital is subdivided into the areas of reception, admittance, labelling, centrifugation and distribution, and is staffed by one medical technologist (MT) and seven paramedic technicians (PMT).The hospital has 720 beds, of which 60 are assigned to the critical patient care unit (ICU, ITU).

### Pre-intervention Lean training

Implementation of the Lean Project, staff training in Lean Health Care methodology and a Kaizen event took place between December 2017 and January 2018.

### Intervention and data collection and analysis

For benchmarking process improvement toward our goal, glucose and haematocrit parameters were selected. Glycaemia was measured using ARCHI-TECT 2000 and ARCHITECT ci8200 systems, whereas haematocrit was measured using an ADVIA 2120i haematology analyser. The primary outcomes measured were turnaround times (TT) for individual testing modalities, defined as the time interval between arrival of the sample at the laboratory and final result.

An analysis was conducted of 6648 data extracted from the LIS system of patients from the medicine (M) and adult emergency (AES) services. The study was approved by the Research Ethics Committee, Universidad Católica de Temuco.

The samples were collected by the phlebotomy team using standardised methods and entered into the intra-laboratory pre-analytical section in the first quarter of 2017 (pre-intervention) and first quarter of 2018 (post-intervention), during working hours.

The database was divided into four groups a) Glucose - Adult Emergency Service (Glucose-AES); b) Glucose - Medicine (Glucose-M); c) Haematocrit - Adult Emergency Service (Haematocrit-AES) and d) Haematocrit - Medicine (Haematocrit-M). To measure the impact of the intervention in line with the routing of the sample through the laboratory, each group was subdivided into four segments: Reception-distribution of samples (R-D), which considers from the reception of samples at the window or via pneumatic delivery system until their distribution within the intra-laboratory pre-analytical section; Distribution-section (D-S), which considers the distribution of samples within the intra-laboratory pre-analytical section until their reception at the areas of Clinical Biochemistry and Haematology; Section-result (S-R), which considers from the arrival of the samples to the analytical areas; and Result-validation (R-V), which comprises from the result of the analysis until its validation.

### Data Analysis

Data analysis was performed using the Graph Pad Prism version 6.0 (San Diego, California USA) statistical software. The Kolmogorov-Smirnov test was used for the normality analysis, while the Mann-Whitney U test was used for independent samples, and *p*<0.05 was considered to be statistically signifi-cant. For this analysis, the time average of year 2017 was considered to be 100% vis-à-vis the times obtained for 2018.

## Results

### »Intra-laboratory pre-analytical phase« pre-intervention workflow

**Figure 1:**
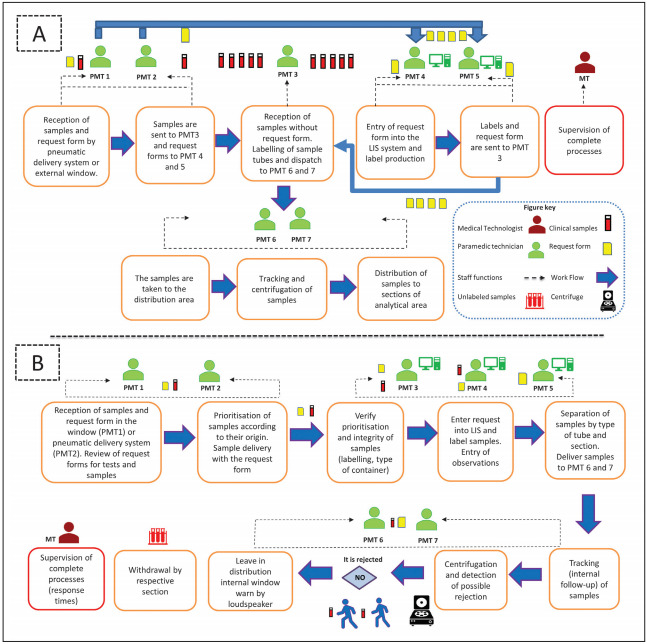
**A** Workflow pre-intervention period and **B.** workflow post-intervention period, route of clinical samples according to staff functions By using the information obtained from our observations and interviews with laboratory personnel (PMT: Paramedic technician; MT: Medical technologist), we redesigned the preanalytic process using Lean management principles. In the process redesign, we were used the same laboratory space and personnel for preanalytic functions, although with a redistribution of functions. Moreover the request form would remain together with the sample during the entire entry into the system and labelling process to generate a continuous and unidirectional flow. The resesigned process map is shown in B.

Figure 1A shows the workflow during the preintervention period in the clinical laboratory. In this period, the samples and request forms arrive at the laboratory reception area via a pneumatic delivery system or directly at the window. After accepting the samples into the section, their admittance, labelling, centrifugation and distribution were carried out. In the pre-analytical section, a discontinuous flow was observed in the route of the samples, regardless of the unit of origin (Figure 1A), giving rise to an accumulation of clinical samples and a delay in recording the data in the LIS system. Moreover, the workflow and staff functions did not allow for adequate coordination to ensure a continuous flow of the samples. The staff functions during the pre-intervention stage, in which these functions were not clearly established, generating a work overload in part of the staff (PMT3) and consequently a delay in the sample flow in certain areas of the laboratory.

### Laboratory infrastructure

During the pre-intervention period, the laboratory was set up in three physical spaces (Sample reception, distribution and centrifugation), where the reception of the samples and their tracking were separated physically, hindering communication between the staff of both sections. Each of these areas were separated by three doors for the entry and exit of staff, sample distribution and separation of tracking and centrifugation areas. In addition, the gas analysing equipment was some distance away from the tracking section, complicating sample flow. During the intervention stage the wall separating the reception area from the distribution area was knocked down leading to an improvement in samples flow and routing, also the arterial gas analysers were relocated, a space and a centrifuge solely for use in priority samples was assigned. One single access and exit route was established for the laboratory staff and the sample distribution door was blocked.

### New workflow post-intervention

After the intervention, a reordering of the laboratory staff was carried out, their functions were reassigned and delimited according to the new work scheme inspired by the Lean methodology, generating rotating work schemes, encouraging teamwork and collaborative work. Although the laboratory staff was maintained. Figure 1B presents the reorganisation of the laboratory workflow (post-intervention period) and the reassignment of PMT functions to generate a continuous and unidirectional flow, establishing priorities depending on the service of origin. In this regard, priority is established by the PMT of the sample reception area in accordance with a list of priorities depending on origin, degree of urgency and type of test, moreover to provide assistance in preanalytical sections and maintenance of necessary consumables for sample taking (PMT1 and PMT2) under the supervision of the Medical Technologist of the unit. Additionally, a distribution of the »digitalisation and labelling« function was carried out depending on the type of sample received (priority, inpatient and culture) for PMT3, PMT4 and PMT5 respectively, optimising the time of entry into the system and analysis of the samples. Moreover, the intervention established that the medical prescription would remain together with the sample during the entire entry into the system and labelling process.

With regard to the centrifugation and distribution process, it is necessary to verify the condition of the sample prior to proceeding with these processes, and samples may be discarded if deemed necessary. Samples meeting the standards are tracked by the system, making it possible to monitor their route through the different sections of the laboratory. Subsequently they are centrifuged in accordance with their priority order, with exclusive-use centrifuges available to deal with urgent samples. Finally, samples are sent to the internal distribution window, where a desk with different colours (representing each section) was implemented to provide a visual aid and speed up the distribution process. Additionally, these paramedical technicians can provide support in window section (reception, admittance / labelling), sample taking, in maintenance of consumables, processing of urine samples and replacement of materials if necessary.

### Kaizen Event

**Figure 2:**
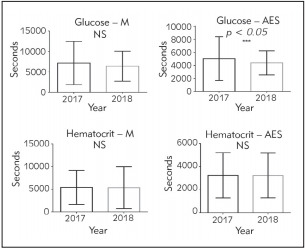
Turnaround times for glucose and haematocrit tests, 2017 versus 2018 at the Medicine (M) and Adult Emergency (AES) services: Comparison charts of turnaround times (TT) for the parameters studied, considering a *p value* < *0.05.* A statistically significant difference was only observed in the Glucose – AES parameter. NS denoted no statistical differences.

Figure 2 presents turnaround times (from the arrival of the sample at the laboratory until its validation) for pre-interventionand post-intervention glucose and haematocrit tests. After theintervention, a significant reduction in the laboratory TT for Glucose-AES was observed (84 to 73 min, 13%, p < 0.05, pre and post). In contrast, although a reduction in the times of the Glucose-M (119 to 106 min, 10.7%, *p* = 0.1530, pre and post), Haematocrit-M (90 to 89 min, 1.2%, *p* = 0.9503 pre and post) and Haematocrit-AES (54 to 53 min, 0.3%, *p* = 0.1468 pre and post), was observed, the results were not statistically significant.

**Figure 3:**
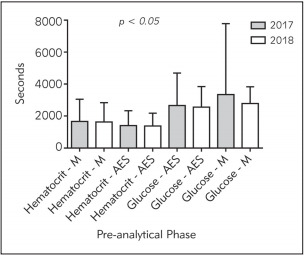
Comparison of turnaround times in haematocrit and glucose tests at the Medicine (M) and Adult Emergency (AES) services, Pre-analytical phase (sections R-D and D-S) Average and standard deviation. NS denoted no statistical differences.

In the analysis by segments, in the intervened phase (pre-analytical RD + DS phase; Figure 3) a significant reduction in the Haematocrit M – AES parameter was observed. On the other hand, when studying the analytical (S-R; Glucose M – AES)and post-analytical (R-V; Haematocrit M – AES) phases (data not shown), a reduction of turnaround times was found (*p<0.05*). However, in S-R; Haematocrit M – AES and in R-V; Glucose M – AES, an increase in TT was observed.

## Discussion

The hospital care system has critical points that affect the expedite and timely attention of users, which are subject to improvement. To address this issue it has been seen that the application of the Lean methodology in health institutions and particularly in clinical laboratories can improve the workflow, increasing patient satisfaction, through an efficient delivery of test analysis results [12] [13] [14] [15]. Lean places emphasis on the elimination of unnecessary steps that provide no value added, in a context of limited financial resources in terms of space, equipment and human capital.

The analysis conducted in the pre-intervention stage made it possible to detect critical points that were modifiable and allowed changes to be made in laboratory staff functions, workflow and infrastructure. In terms of workflow at the laboratory, the adjustments led to the establishment of a continuous and unidirectional flow (Figure 1B), giving priority to the work in accordance with the service of origin. Thus, during the reception of samples, it was established that PMT1 and PMT2 are to examine their condition and check that the request form contains patient data, origin, test required, informed consent when necessary and signature of the requesting doctor. Noncompliance with these requirements are to be recorded and notified as cause for rejection. During the digitalisation of the samples in the LIS system, a new control point was established in order to check that the information contained in the test requirement and the sample are complete. In the event that a sample is unsuitable, this observations to be entered into the LIS system, noting the criterion for rejection and requesting a new sample. The results show that the intervention inspired on the Lean methodology significantly reduced the turnaround times in the glucose parameter issuing from the adult emergency service.

To see whether intervention in the pre-analytical phase would have an impact on other sections of the laboratory, the routing segments of the samples were analysed in terms of reception-distribution (R-D); distribution-section (D-S), section-result (S-R) and resultvalidation (R-V), showing that the intervention reduced the turnaround time in the intra-laboratory pre-analytical phase (Figure 3). However, these same results were not observed in the analytical and postanalytical phases (data not shown), where in some cases the turnaround time increased.

In a similar manner to the Moron-Castaneda et al. [16] study, a reduction in analysis and result time was obtained, lowering turnaround times in up to 9 minutes and improving client satisfaction.

Although the analyses per segment showed that there is a reduction in response times, inS-R; Haematocrit M–AES and R-V; Glucose M–AES an increase in response times was observed, suggesting that it is necessary to systematise and address the entire model, including the processes in the analytical and post-analytical phases. The results observed could be explained in part by the fact that the improvement in the pre-analytical phase gave rise to a greater workflow toward the subsequent areas (analytical and post-analytical), an also because there was an increase in the demand for test requests in 2018. The above supports the idea of establishing improvements in the analytical phase in this area of the laboratory. The evidence shows that optimising workflow in all its areas via a management model would make it possible to optimise the process, giving rise to a continuous flow for both specimens and operator [14].

The re-design in the organisation of the laboratory structure made it possible to optimise resources, improve workflow dynamics and needs, taking into consideration that no single organisation modality has been implemented. In terms of staff satisfaction, the feedback was positive, showing that the intervention contributed to better define the functions of each member of the team. Limitation to the study is that it was carried out in just one institution and the results could prove not to be applicable in other clinical laboratories with very different workflows. Lastly, user satisfaction was not assessed, which would make it possible to establish new criteria to be modified in order to achieve continuous improvement of these units.

## Conclusion

The results show that the Lean methodology is applicable to improve the pre-analytical phase in the laboratory, expediting the turnaround time of the delivery of clinical test results. This study reinforces the usefulness of applying the Lean model to improve the efficiency of diagnostic processes, and was one of the first experiences in Chile in the use of this methodology in a clinical laboratory setting.

The intervention carried out in this study was achieved with minimum financial investment (except for the structural modifications), by means of optimising teamwork and assertive communication, with no staff changes made, and contributing to install a culture of continuous improvement in the laboratory.

## Acknowledgements

The authors wish to express their appreciation to the work team at the clinical laboratory of the Dr. Hernán Henríquez Aravena Hospital of Temuco and to the Roche Diagnostics® Company for the staff training process. Research funding: Supported by FEQUIP2018-PL-06 and VIPUCT N°2016PF PL-04 grant from the Vicerrectoría de Investigación y Postgrado, Universidad Católica de Temuco.

## Conflict of interest statement

All the authors declare that they have no conflict of interest in this work.

## List of abbreviations

LIS, Laboratory Information System; MT, medical technologist; PMT, and seven paramedic technicians; ICU, ITU, critical patient care unit; TT, turnaround times; M, medicine services; AES, adult emergency services; R-D, Reception-distribution of samples; D-S, Distribution-section; S-R, Section-result; R-V, Result-validation.
